# Miscellaneous

**Published:** 1863-02

**Authors:** 


*Miscellaneous.*—The Cincinnati *Lancet & Observer* confounds the CHICAGO MEDICAL JOURNAL with a city cotemporary. It says it has not received a number of this journal for two months. Singularly enough, in the same No. of the *Lancet*, is noticed a case reported in this journal the month previous, viz: Dr. Holmead's case of Cœsarean Section. It argues that this journal is delayed in publication in consequence of pecuniary embarrassment. This is by no means the case, and has at no time been. The delay of the Jan. No. was simply on account of sickness of the editor. We issue the JOURNAL during the month named on the title page, not imitating the secular monthlies by anticipating the month. The February No. is delayed one week from the same cause.

Our thanks are due Dr. Wardner for copies of the *Mound City Hospital* regulations.

In a recent case of trial for mal-practice, where it was attempted to show that *paralysis* resulted from *inhalation of Chloroform*, the jury found a verdict for the defendant. Prof. S. D. Gross, in his testimony on cross-examination, said: "Chloroform, like many other agents which a physician is obliged to use, is dangerous; so is laudanum, &c.

*Question.* If improperly used would it not produce paralysis?

*Answer.* No, sir.

*Ques.* Would it be improper to continue the use of chloroform after a patient has resisted its influence for nearly three quarters of an hour?

*Ans.* No, sir; I should continue for five hours, until I accomplished my object; I have taken chloroform myself; a patient who resists, only proves that he has not taken enough."

All of which may perhaps be steadfastly believed; nevertheless it is difficult to understand why this most potent of anæsthetics may not produce local disturbances which may ultimate in paralysis. The patient in this case had been injured some time previous by a fall on the head. This accident probably determined the special effect—paralysis—culminating on the use of chloroform.

Medical Director J. V. Z. Blaney, with Surgeons Stoker and Huntington, have been designated as a board to examine into the professional, physical and moral qualifications of such medical officers as the authorities of the Seventh Army Corps, (Department of Virginia,) may direct. We extract from the detailing order:

"While many are discharging their responsible duties in a creditable manner, a few have obtained positions in the army without the proper qualifications. The well-merited reputation of the efficient ought not to be compromised by the incompetent. The welfare of the sick and wounded and the best interests of the service demand that all such should be promptly removed."

We have it from the best authority, that of the large number who have presented themselves for examination before the Board at Washington for examining candidates for Brigade Surgeons, (now Surgeons of Volunteers), *sixty per cent.* have been rejected. There are now three hundred vacancies in the two grades. We suggest to some of the graduates of Rush Medical College to present themselves before the national board. We have heard it intimated that the Faculty of Rush are willing that their graduates should go before the national examining board, or any other board not committed, shoulder deep, to the interests of some petty rivalry or clique.

Dr. Morton (with a considerable proportion of the alphabet prefixed to the surname) is again before Congress with a petition to be rewarded for introducing anæsthesia in surgical operations to the world.

